# Factors contributing to the rising prevalence of waterpipe smoking dependence among university students: a cross-sectional study

**DOI:** 10.1186/s12909-024-05156-0

**Published:** 2024-02-20

**Authors:** Beesan Maraqa, Zaher Nazzal, Noor Abu Baker, Heyam Khatib, Marwa Zeyad, Omar Aburayyan

**Affiliations:** 1 Department of Medicine, Faculty of Medicine, Hebron University, Hebron, Palestine; 2 Primary HealthCare, Ministry of Health, Hebron, Palestine; 3 Community Medicine, Department of Medicine, Faculty of Medicine and Health Sciences, An-Najah National University, Nablus, Palestine; 4 Department of Medicine, Faculty of Medicine and Health Sciences, An-Najah National University, Nablus, Palestine; 5 Department of Medicine, Faculty of Medicine and Health Sciences, Al-Quds University, Al-Quds, Palestine

**Keywords:** Normative believes, Dependence, Wastepipe smoking, University students

## Abstract

**Introduction:**

Waterpipe smoking (WPS) has been associated with a variety of adverse health effects, consequences, and symptoms of nicotine dependence. This study aims to determine the prevalence of WPS dependence among Palestinian university waterpipe smoking students, their normative beliefs about WPS, and their relationship to dependence.

**Methods:**

A cross-sectional study of current WPS university students at five major Palestinian universities was conducted from September to December 2022. Participants were recruited using convenient sampling, and data collection was carried out via an interviewer-administered questionnaire. We assessed students’ WPS dependence using the Lebanese Waterpipe Dependence Scale. We used the Poisson regression model with robust variance to analyze factors independently associated with high WPS dependence.

**Results:**

The study included 746 current WPS university students. Results revealed a high prevalence of WPS dependence, with 69.4% (95%CI: 66.0-72.7%) exhibiting high dependence. Factors contributing to high WPS dependence included dual cigarette smoking (aPR: 1.18; 95%CI: 1.12–1.25), studying medical sciences (aPR: 1.13; 95%CI: 1.10–1.18), friends’ approval of WPS (aPR: 1.25; 95%CI: 1.17–1.34), daily WPS frequency (aPR: 1.98; 95%CI: 1.39–2.23), spending more than 50% of daily allowance on WPS (aPR: 1.37; 95%CI: 1.10–1.64), and morning WPS sessions (aPR: 1.97; 95%CI: 1.31–2.27). The study highlighted the influence of social factors, such as peers’ approval and perceived prevalence, on WPS dependence.

**Conclusions:**

WPS dependence is prevalent among university students, and it is associated with morning WPS, dual smoking, and increased WPS frequency. Notably, peer and cultural factors are essential primary motivators. As a result, it is critical to incorporate WPS considerations into Palestine’s antismoking health promotion program. Therefore, it is vital to incorporate WPS into the Palestinian antismoking health promotion policy, and the health education of adolescents regarding the dangers of WPS should coincide with antismoking initiatives.

**Supplementary Information:**

The online version contains supplementary material available at 10.1186/s12909-024-05156-0.

## Introduction

Waterpipe is a popular smoking device that allegedly delivers more nicotine than ordinary cigarettes; one head has the nicotine equivalent of seventy cigarettes [1]. Waterpipe smoking (WPS) has surged globally, especially among younger and university students, with the Eastern Mediterranean Region and Europe reporting the highest prevalence, and it is more common among youth than adults [2].

The rate of smoking in Palestine is one of the highest in the region. Studies show a range of prevalence, with figures indicating it varies from 20.0 to 35.0% in the general population and from 35 to 56% among university students [3–5]. In the context of WPS, around one-fourth of university students are current users, surpassing the percentage of cigarette smokers [4, 6]. This trend is reinforced by the perception that WPS is considered less harmful and more socially acceptable compared to cigarette smoking [7].

WPS among university students can potentially negatively affect academic success and well-being. Studies suggest that it may adversely affect cognitive function and concentration, presenting obstacles to academic performance [8]. Increased rates of WPS have been associated with lower levels of academic achievement [4]. Furthermore, similar to cigarette smoking, WPS has been linked to several adverse health effects, including lung cancer, oral cancer, cardiovascular diseases, and respiratory diseases [9–11]. Also, it has been linked to a variety of mental health conditions, including depression and anxiety [12], and it has also been demonstrated that more prolonged and more frequent WPS sessions are addictive [13].

During a WPS episode, the user obtains active doses of nicotine that are known to produce dependence when taken regularly [14], which makes many smokers experience withdrawal and other symptoms resulting from nicotine dependence [15, 16]. Research on WPS dependence has predominantly focused on adult smokers; however, there is an increasing body of evidence suggesting that adolescents also display symptoms of dependence. Some studies have indicated that university students exhibited high WPS dependence [17–19].

WPS dependence is attributed to several factors, including a higher frequency of smoking sessions, monthly spending on tobacco products, concurrent usage of traditional smoking, peer and societal influence, and parental disapproval of smoking [19–21]. It can lead to adverse outcomes and behaviors, including motivations for engaging in risky behaviors [17], depressive symptoms [22], and eating disorders [23]. Normative beliefs are thought to influence people’s intentions and behaviors significantly. It has been frequently employed in the study of cigarettes and WPS [24], but its potential relationship with WPS dependency has received little attention [21].

Despite indications that WPS dependence is increasing, there still needs to be more research on the subject, notably among university students in Palestine and the Middle East Region. Therefore, this study is anticipated to address this crucial gap by examining the prevalence of WPS dependence among university students and exploring associated factors such as normative beliefs, societal influence, parental disapproval, and expenditure on tobacco products. The findings of this study help inform policymakers and health professionals to develop targeted preventive measures and emphasize the importance of proactive interventions to control the rising trend of WPS and its potential consequences.

## Methods and materials

### Study design and population

We conducted a cross-sectional study with Palestinian university students from September to December 2022. The study occurred at five major Palestinian universities: An-Najah National University, Birzeit University, Arab American University, Al-Quds University, and Hebron University. These institutions are the largest in the West Bank, collectively representing its three main regions: the northern, southern, and central areas. The study population consisted of current WP-smoking university students who smoked at least one waterpipe per month. The inclusion criteria comprised full-time undergraduate students enrolled at the specified universities.

### Sample size and sampling

A sample size of at least 660 students was calculated as adequate for achieving the study’s objectives. It was determined using the formula for descriptive studies: [n = [DEFF * Np(1 p)]/ [(d2/Z21/2*(N 1) + p * (1 p)], where the estimated proportion of university students with high WPS dependence is 50%, with a 95% confidence level, a 4% confidence limit, and a design effect of 1. To account for incomplete questionnaires, the sample size was increased by 15%, yielding a final sample size of 760 students.

It was challenging to select a random sample due to the constraints imposed by the universities in not providing the researchers with a list of students enrolled in each university. As a result, we chose students conveniently by communicating with them during breaks and between classes, carefully ensuring a heterogeneous and representative sample across all academic disciplines, age groups, and genders. The Institutional Review Board of An-Najah National University approved the study [Ref #: Med. March. 2022/22]. All students were approached and invited to participate voluntarily, with the assurance that the information acquired would be kept confidential. Students who agreed to take part in the study signed informed consent.

### Measurement tool

The study’s measuring tool was a three-part, interviewer-administered questionnaire developed by the research team. The first part assessed the students’ sociodemographic factors, including age, gender, residence, living situation, physical activity, and active smoking history, as well as questions about their families’ and friends’ smoking histories. Residency was classified as urban for students residing in Palestinian cities and rural for those living in Palestinian villages. Physical activity was defined as 150 min of moderate-intensity aerobic physical activity or at least 75 min of vigorous-intensity aerobic physical activity throughout the week. Those who smoked cigarettes on one or more days during the past 30 days were considered active smokers.

In the second section, the Lebanese Waterpipe Reliance Scale (LWDS-11), developed and validated by Salameh et al. [25], assessed WPS dependence. It consists of eleven items measured on a four-point Likert scale ranging from 0 to 3 and covers four subscales: nicotine dependency, negative reinforcement, psychological craving, and positive reinforcement. The scale was evaluated among different populations and has good psychometric properties to measure WPS dependence among university students [21, 26]. The total LWDS-11 score ranges from 0 to 33, with a score of > 10 indicating high WPS dependence [25]. The internal consistency (Cronbach’s Alpha) of the LWDS-11 in this study was 0.810.

The third section of the questionnaire assessed the students’ normative beliefs regarding WPS. It included ten items from previous studies on cigarettes and WPS [21, 27]. Four items assessed students’ perceptions about whether certain successful or elite members of society were likely to be waterpipe smokers. They were assessed on a 4-point Likert scale (strongly agree/agree/disagree/strongly disagree) before being reduced to binary variables: perceived normal (Yes or No). Two items addressed their perception that their parents and friends disapproved of their WPS and considered it a bad habit. Again, they were assessed on a 4-point Likert scale (strongly agree/agree/disagree/strongly disagree) before being reduced to binary variables: perceived disapproval (Yes or No). The final four items in section three assessed the “perceived prevalence,” a belief about most of a social group’s behavior. The more prevalent a behavior is perceived, the more likely an individual will accept it as usual. The students were asked to estimate the proportion of persons who smoke waterpipe in Palestine, their cities, their universities, and their surroundings on a scale of 0 to 100 in 10-point increments before the estimations were reduced to normal, high, and very high. Before the initial study, the questionnaire was pilot-tested on 30 students, and three experts revised it in the field to enhance its validity and reliability. The research team (NB, HK, MZ, and OA) reached out to students at selected universities and, upon receiving their voluntary consent to participate, conducted interviews during which the questionnaire was completed.

### Data analysis

The data were analyzed using Statistical Package for the Social Sciences version 26 (IBM Corp., Armonk, NY, United States) and presented as frequencies and percentages for categorical variables and as means and standard deviations (SD) for continuous variables. We calculated the prevalence of high WPS dependence and its 95% Confidence Intervals (95%CI). A chi-squared test and an independent t-test were used to compare high WPS dependence between groups. Multivariable analysis was employed using the Poisson regression model with robust variance to adjust for confounders and analyze factors independently associated with high WPS dependence. The findings were reported as an adjusted prevalence ratio (aPR) with a 95%CI. We selected this model because odds ratios calculated in cross-sectional studies using logistic regression may overestimate prevalence ratios when the outcome is common [28, 29]. All factors with significant associations in bivariate analysis and those determined to be very relevant in the literature were included in the model. Statistical significance was defined as p-values less than 0.05.

## Results

Out of 820 university students invited, 760 (92.5%) participated, and 746 (98.2%) with complete LWDS-11 responses were analyzed. The mean age of the participants was 20.5 ± 1.6 years, and 59.1% were males. Most (80.3%) lived with their families, and 49.5% were from Humanities faculties. Of the participants, 69.4% (95% CI: 66.0-72.7%) showed high WP dependence (Table 1).

**Table 1:** Background characteristics of university students, along with the difference between high and low WPS dependence groups (*n* = 746)

	Total	WPS Dependence		P value*
	Frequency (%)	High	Low	
**Age** *(Mean ± SD)*	20.5 ± 1.6	20.6 ± 1.6	20.3 ± 1.7	0.141
≤ 20 years	431 (57.8%)	284 (65.9%)	147 (34.1%)	0.016
> 20 years	315 (42.2%)	234 (74.3%)	81 (25.7%)	
***Gender***				< 0.001
Male	441 (59.1%)	333 (75.5%)	108 (24.5%)	
Female	305 (40.9%)	185 (60.7%)	10 (39.3%)	
***Residency***				
Urban	413 (55.7%)	284 (68.8%)	129 (31.2%)	0.631
Rural	329 (44.3%)	232 (70.5%)	97 (29.5%)	
***Current living place***				
With family	597 (80.3%)	406 (68.0%)	191 (32.0%)	0.100
With students	91 (12.3%)	72 (79.1%)	19 (20.9%)	
Alone	55 (7.4%)	38 (69.1%)	17 (30.9%)	
***Family size***				0.811
≤ 5	399 (53.7%)	279 (69.9%)	120 (30.1%)	
More than 5	344 (46.3%)	237 (68.9%)	107 (31.1%)	
***Time spent with friends a day***				0.085
Less than 2 h	90 (12.1%)	55 (61.1%)	35 (38.9%)	
2- 5 h	361 (48.4%)	284 (68.7%)	113 (31.3%)	
More than 5 h	294 (39.5%)	215 (73.1%)	79 (26.9%)	
***Physical activity***				0.487
None	201 (26.9%)	139 (69.2%)	62 (30.8%)	
Moderate	388 (52.0%)	264 (68.0%)	124 (32.0%)	
High	157 (21.0%)	115 (73.2%)	42 (26.8%)	
***College***				
Humanities	367 (49.5%)	248 (67.6%)	119 (32.4%)	0.092
Sciences-nonmedical	200 (27.0%)	132 (66.5%)	67 (33.5%)	
Sciences-medical sciences	174 (23.5%)	133 (75.9%)	42 (24.1%)	

^*^ *Chi-squared test and independent t-test*

**Fig. 1:**
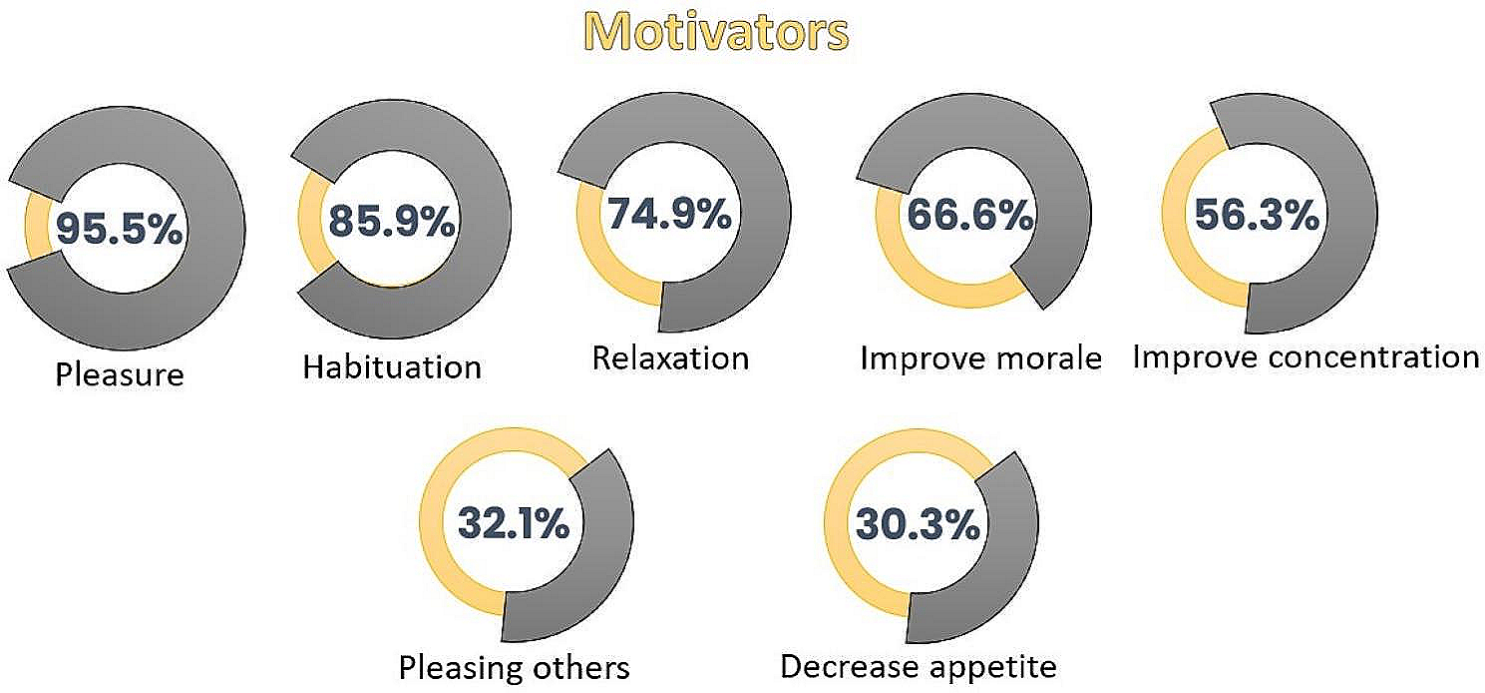
Motivators of WPS among palestinian university students

Almost half of students engaged in WPS at least three times weekly, with 15.1% being regular morning smokers. Additionally, 25.4% allocated 11–50% of their daily allowance to WPS. Around one-third of the respondents were dual smokers, and the majority of students’ friends were either cigarette or waterpipe smokers (Table 2). Students indicated enjoyment and habit were their main reasons for engaging in WPS (Fig. 1).

**Table 2:** Tobacco smoking characteristics of university students, along with the difference between high and low WPS dependence groups (*n* = 746)

	Total	WPS Dependence		P value*	
	Frequency (%)	High	Low		
***Cigarette smoker***					
Yes	253 (33.9%)	192 (75.9%)	61 (24.1%)	0.007	
No	492 (66.1%)	326 (66.1%)	167 (33.9%)		
***Vape smoker***					
Yes	238 (31.9%)	176 (73.9%)	62 (26.1%)	0.067	
No	508 (68.1%)	342 (67.3%)	166 (32.7%)		
***Number of WPS per week***					
< 1	185 (24.8%)	54 (29.2%)	131 (70.8%)	< 0.001	
1–2 times	201 (26.9%)	144 (71.6%)	57 (28.4%)		
3–6 Times	211 (28.3%)	178 (84.4%)	33 (15.6%)		
≥ 7 times	149 (22.0%)	142 (95.3%)	7 (4.7%)		
***Percent of daily allowance spent on WPS***					
≤ 10% of daily allowance	468 (62.7%)	271 (57.9%)	197 (42.1%)	< 0.001	
11–50% of daily allowance	189 (25.4%)	161 (85.2%)	28 (14.8%)		
> 50% of daily allowance	89 (11.9%)	86 (96.6%)	3 (3.4%)		
***Time in the day WPS***				< 0.001	
Always morning	37 (5.0%)	35 (94.6%)	2 (5.4%)		
Morning more than the afternoon	75 (10.1%)	63 (84.0%)	12 (16.0%)		
Afternoon more than morning	277 (37.2%)	228 (82.3%)	49 (17.7%)		
Always Afternoon	355 (47.7%)	191 (53.8%)	164 (46.2%)		
***Cigarette smoking father***					
Yes	397 (53.2%)	276 (69.5%)	121 (30.5%)	0.954	
No	349 (46.8%)	242 (39.3%)	107 (30.7%)		
***Waterpipe smoking father***					
Yes	128 (17.2%)	104 (81.3%)	24 (18.8%)		
No	618 (82.8%)	414 (67.0%)	204 (33.0%)	0.001	
***Cigarette smoking mother***					
Yes	73 (9.8%)	49 (67.1%)	24 (32.9%)	0.689	
No	673 (90.2%)	469 (69.7%)	204 (30.3%)		
***Waterpipe smoking mother***					
Yes	128 (17.2%)	97 (75.8%)	31 (24.2%)	0.092	
No	618 (82.8%)	421 (68.1%)	197 (31.9%)		
***Cigarette smoking friends***					
Yes	440 (59.1%)	316 (71.8%)	124 (28.2%)	0.105	
No	304 (40.9%)	201 (66.1%)	103 (33.9%)		
***Waterpipe smoking friends***					
Yes	517 (69.5%)	373 (72.1%)	144 (27.9%)	0.020	
No	227 (30.5%)	144 (63.4%)	83 (36.6%)		

^*^ *Chi-squared test*

Students who exhibited a high WPS dependence showed notably heightened perceptions regarding the likelihood of successful, popular, and famous individuals being waterpipe smokers (P value < 0.05 for all). Moreover, they demonstrated a significantly reduced perception that their peers disapproved of their WPS and considered it an undesirable habit (P value < 0.05 for all). The perceived prevalence of WPS was high, with more than 80% of students perceiving WPS as high or very high in their cities, universities, and surroundings (Table 3).

**Table 3:** Students’ normative beliefs of waterpipe smoking and the difference between high and low WPS dependence groups (*n* = 746)

	Total	WPS Dependence		P-value*
Statements	Frequency (%)	High	Low	
***Successful people smoke***				
Yes	219 (29.4%)	179 (81.7%)	40 (18.3%)	< 0.001
No	525 (70.6%)	337 (64.2%)	188 (35.8%)	
***Cool people smoke***				
Yes	266 (35.8%)	200 (75.2%)	66 (24.8%)	0.010
No	477 (64.2%)	315 (66.0%)	162 (34.0%)	
***Rich people smoke***				
Yes	226 (30.4%)	165 (73.0%)	61 (27.0%)	0.153
No	518 (69.6%)	351 (67.8%)	167 (32.2%)	
***My idols smoke***				
Yes	225 (30.2%)	171 (76.0%)	54 (24.0%)	0.011
No	519 (69.8%)	346 (66.7%)	173 (33.3%)	
***My family sees WPS as a bad habit***				
Yes	584 (78.7%)	400 (68.5%)	184 (31.5%)	0.299
No	158 (21.3%)	115 (72.8%)	43 (27.2%)	
***My friends see WPS as a bad habit***				
Yes	252 (33.8%)	158 (62.7%)	94 (37.3%)	0.004
No	494 (66.2%)	360 (72.9%)	134 (27.1%)	
***Perceived WPS in Palestine***				
Normal	51 (6.8%)	40 (78.4%)	11 (26.6%)	0.255
High	144 (19.3%)	103 (71.5%)	41 (28.5%)	
Very high	551 (73.9%)	375 (68.1%)	176 (31.9%)	
***Perceived WPS in their cities***				
Normal	76 (10.2%)	53 (69.7%)	23 (30.3%)	0.782
High	125 (16.8%)	90 (72.0%)	35 (28.0%)	
Very high	545 (73.0%)	375 (68.8%)	170 (31.2%)	
***Perceived WPS in their universities***				
Normal	82 (11.0%)	62 (75.6%)	20 (24.4%)	0.426
High	173 (23.2%)	120 (69.4%)	53 (30.6%)	
Very high	491 (65.8%)	336 (68.4%)	155 (31.6%)	
***Perceived WPS in their surroundings***				
Normal	128 (17.2%)	93 (72.7%)	35 (27.3%)	0.317
High	209 (28.0%)	137 (65.6%)	72 (34.4%)	
Very high	409 (54.8%)	288 (70.4%)	121 (29.6%)	

^*^ *Chi-squared test*

### Multivariable analysis

We used Poisson multivariable regression with robust variance to analyze variables related to the study outcome. Students studying medical sciences (aPR 1.13; 95%CI: 1.10–1.18), those who are dual cigarette smokers (aPR 1.18; 95%CI: 1.12–1.25), and students whose friends approve WPS (aPR 1.25; 95%CI: 1.17–1.34) were more likely to have high WPS dependence. Students who smoked waterpipe once daily were nearly twice as likely as those who smoked less than once (aPR 1.98; 95%CI: 1.39–2.32), while students who spent more than 50% of their daily allowance on WPS were 1.4 times more likely to have high WPS dependence (aPR 1.37; 95%CI: 1.10–1.64) than students who spent less than 10% of their daily allowance on WPS. Furthermore, students who consistently engaged in morning WPS were nearly twice as prone to experiencing high WPS dependence (aPR 1.97; 95%CI: 1.31–2.27) compared to those who consistently smoked in other patterns (Table 4).

**Table 4:** Multivariable Poisson regression (with robust variance) analysis of variables related to high WPS dependence

	**Prevalence Ratio**	**95%CI**	**aP value**	
***Age****(Ref*: ≤20 years*)*	0.996	0.98–1.01	0.509	
***Gender*** *(Ref: Female)*	0.97	0.93–1.12	0.229	
***Time spent with friends a day*** *(Ref: Less than 2 h)*				
2–5 h	0.99	0.93–1.11	0.843	
More than 5 h	1.12	0.97–1.47	0.497	
***College****(Ref*: Humanities*)*				
Sciences-nonmedical	0.98	0.94–112	0.395	
Sciences-medical sciences	1.13	1.10–1.18	0.037	
***Cigarette smoker*** *(Ref: No)*	1.18	1.12–1.25	0.008	
***Vape smoker*** *(Ref: No)*	1.05	0.97–1.14	0.795	
***Number of WPS per week*** *(Ref: Less than once)*				
1–2 times	1.28	1.20–1.56	< 0.001	
3–6 Times	1.43	1.30–1.64	< 0.001	
≥ 7 times	1.98	1.39–2.32	< 0.001	
***Percent of daily allowance spent on WPS****(Ref*: ≤10% of daily allowance*)*				
11–50% of daily allowance	1.18	1.05–1.27	< 0.001	
> 50% of daily allowance	1.37	1.10–1.64	< 0.001	
***Time in the day WPS****(Ref*: Always afternoon*)*				
Always morning	1.97	1.31–2.27	< 0.001	
Morning more than afternoon	1.47	1.27–1.69	< 0.001	
Afternoon more than morning	1.28	1.20–1.56	< 0.001	
***Waterpipe smoking father*** *(Ref: No)*	1.04	0.97–1.08	0.486	
***Waterpipe smoking mother*** *(Ref: No)*	1.03	0.98–1.07	0.216	
***Waterpipe smoking friends*** *(Ref: No)*	1.02	0.97–1.05	0.605	
***Normative beliefs***				
* Successful people smoke waterpipe (Ref: No)*	1.16	0.99–1.23	0.052	
* Cool people smoke waterpipe (Ref: No)*	0.99	0.95–1.15	0.575	
* My idols smoke waterpipe (Ref: No)*	1.13	0.99–1.27	0.073	
* My friends disapprove WPS (Ref: Yes)*	1.25	1.17–1.34	0.019	

*CI*: *confidence interval;**** aP value***: *adjusted P value*

## Discussions

WPS has become increasingly widespread recently, particularly among younger people. In Palestine, university students’ WPS exceeds that of cigarettes. Smells, sounds, and tastes have been identified as essential motivators for WPS university students [4]. According to our findings, the primary drivers of WPS are pleasure, relaxation, and habituation. Both positive reinforcement, such as enjoyment and socialization, and negative reinforcement, like using WPS to calm nerves, are linked to increased nicotine dependence or psychological craving [30]. WPS is found to have the same dependence and harmful effects as tobacco [31]. The results of our study revealed that 69.4% of the participants display high WPS dependence, aligning with comparable findings in Lebanon [18], where 69.5% of university students exhibited high WPS dependence, and in Bangladesh [19], where 72% of university students were classified as having high WPS dependence. In Jordan’s universities, 54.1% of WPS students reported moderate to high nicotine dependence [32]. This high dependence rate carries significant implications for health policy. Like cigarette tobacco policies, policies addressing WPS should be introduced with robust measures aimed at behavioral change, including smoking-free legislation and health warning labeling [33].

The social environment significantly influences nicotine dependence at the population level, particularly in the context of WPS [34]. The influence of peers has a substantial impact on WPS dependency; our data reveal that students whose friends approve of WPS are more likely to have high dependence. The results about how others’ approval or disapproval of WPS affects people, including family and friends, and how that affects their dependence on WPS could be used as the basis for interventions that try to change how people think about WPS. Previous evidence has shown that interventions grounded in normative influences can effectively bring about behavioral changes, including smoking cessation and decreased alcohol and drug consumption [24].

The percentage of daily allowance (pocket money) spent on WPS was positively associated with the WPS dependence score. These individuals are predominantly from the lower socioeconomic class [35], thereby contributing to the increasing poverty in the community. A subgroup analysis revealed that all students spending more than 50% of their daily allowance on WPS exhibited high waterpipe dependence. However, it is deemed unacceptable for college students to use their funds for WPS. The study suggests a concerning trend of college students allocating funds to WPS, and similar findings were reported in other universities, linking WPS dependence scores with monthly tobacco product expenditure [19].

Frequency of usage and concurrent use of many tobacco products are surrogate indicators of a high rate of nicotine use. Almost a quarter of our university students use the waterpipe at least once daily. Higher WPS frequency is strongly associated with dependence, with those smoking seven or more times per week being almost twice as likely to exhibit high dependence. Consistent with these findings, a higher risk of nicotine dependence has been associated with increased use frequency, number of waterpipes smoked, and longer smoking sessions [15, 25, 36]. The concurrent use of cigarettes and waterpipes raises the risk of nicotine dependence [37]. Our results showed that 33.9% of the university students are dual smokers (waterpipe and cigarette), and they are 1.18 times more likely to have high WPS dependence. The findings suggest that monitoring the frequency of WPS, along with the concurrent use of cigarettes and waterpipes, can serve as valuable indicators of high nicotine use and dependence.

Compared to WPS only in the afternoons, WPS in the morning strongly indicates a high dependence. The amount of time it takes to smoke one’s first cigarette after waking up has been used as the best single-item measure of nicotine dependence in several studies [38], and it has also been linked to an increased risk of certain diseases such as lung cancer and COPD [39]. These findings are critical for healthcare professionals to consider when counseling smokers about health risks, and they can serve as a foundation for evaluation, effective management, and follow-up.

Students’ perception of the prevalence of WPS in the general population did not correlate with a high WPS dependence. Nevertheless, a notable finding was that a significant majority of WPS students, exceeding 80%, perceive the prevalence of waterpipe smoking as high or very high within their cities, universities, surrounding areas, and even across Palestine. This aligns with the actual situation in Palestine, as indicated by the first specialized survey on smoking and tobacco consumption in 2021. The survey reported an increase in the overall prevalence of smoked tobacco among adults aged 18 years and above to 31%, compared to 23% in 2010. Moreover, exclusive use of WPS, without other tobacco types, constituted approximately 21% of the total current smokers [5]. These findings highlight the importance of recognizing the social influences individuals experience during key phases like testing, initiation, and maintenance of tobacco use. It implies that achieving a tobacco-free standard among adolescents requires a proactive approach to counteracting any messages that promote tobacco use.

Another important finding is that students in the field of medical sciences were more likely to show high WPS dependence compared to their peers from other academic disciplines. This trend may be attributed to the increased vulnerability to nicotine dependence among regular waterpipe smokers. Existing literature highlights that a substantial portion of health sciences university students in the region engage in WPS, with up to 50% being waterpipe smokers [40, 41]. These findings are concerning because medical science students are expected to have excellent knowledge of the risks and effects of tobacco use and to communicate it to their patients in their future careers. These findings have positive implications and demonstrate that relying just on knowledge is insufficient to reduce smoking prevalence. It emphasizes the importance of incorporating evidence-based interventions into the design of programs targeted at addressing and reducing the prevalence of smoking among young adults.

Our study has limitations that should be considered when interpreting its findings. Firstly, despite our best efforts to diversify the sample by selecting participants from various locations, times, and educational days within each university, the non-random nature of the sample might limit its representativeness for the entire WPS student population in Palestine. Secondly, our study exhibited a higher proportion of male participants than females. However, this gender distribution aligns with the known prevalence of WPS, which is more commonly observed among males than females, given that our study exclusively involved WPS students. Thirdly, because the study was conducted using an interviewer-administered questionnaire, the possibility of social desirability bias and underreporting cannot be ruled out in this study, even though no identifying information was collected and all collected data was kept confidential. Lastly, due to the necessity of maintaining a manageable questionnaire length to enhance student acceptance and response rates, not all variables associated with WPS dependence could be included in the study.

## Conclusions

WPS dependence is highly prevalent among Palestinian university students, which poses a substantial health risk, just like other tobacco products. Morning WPS is most frequently linked to dependence. Peer influence and societal factors are the primary motivators. The study’s findings provide valuable understanding for policymakers, health educators, and public health practitioners, offering a basis for developing targeted strategies to combat WPS dependence among university students. The results underscore the importance of addressing societal norms, peer influences, and individual behaviors to reduce WPS prevalence and its associated health risks. As the prevalence of hookah smoking among females rises, we recommend that future studies compare both genders to gain a better understanding of this emerging trend.

### Electronic supplementary material

Below is the link to the electronic supplementary material.

Supplementary Material 1: The questionnaire

## Data Availability

The dataset supporting the conclusions of this article is included within the article and its additional files.
